# Exploring Comorbidities in Adolescent and Young Adults with Hypermobile Ehlers–Danlos Syndrome with and without a Surgical History: A Preliminary Investigation

**DOI:** 10.3390/children10091562

**Published:** 2023-09-16

**Authors:** Haley Gagnon, Claire E. Lunde, Ziyan Wu, Eduardo N. Novais, David Borsook, Christine B. Sieberg

**Affiliations:** 1 Biobehavioral Pain Innovations Laboratory, Department of Psychiatry & Behavioral Sciences, Boston Children’s Hospital, Boston, MA 02115, USA; hegagnon@bu.edu (H.G.); claire.lunde@childrens.harvard.edu (C.E.L.); ziyan.wu@childrens.harvard.edu (Z.W.); 2 Pain and Affective Neuroscience Center, Department of Anesthesiology, Critical Care & Pain Medicine, Boston Children’s Hospital, Boston, MA 02115, USA; 3 Nuffield Department of Women’s & Reproductive Health, University of Oxford, Oxford OX3 9DU, UK; 4 Department of Orthopedic Surgery, Boston Children’s Hospital, Boston, MA 02115, USA; eduardo.novais@childrens.harvard.edu; 5 Department of Orthopedic Surgery, Harvard Medical School, Boston, MA 02115, USA; 6 Departments of Psychiatry and Radiology, Massachusetts General Hospital, Boston, MA 02114, USA; dborsook@mgh.harvard.edu; 7 Department of Anesthesiology, Harvard Medical School, Boston, MA 02115, USA; 8 Department of Psychiatry, Harvard Medical School, Boston, MA 02115, USA

**Keywords:** hypermobile Ehlers–Danlos syndrome (hEDS), chronic pain, medical comorbidities, surgery

## Abstract

Ehlers–Danlos Syndrome (EDS) is a rare disease affecting the skin, joints, vasculature, and internal organs. Approximately 85% of those affected are categorized as the hypermobile type (hEDS), which is associated with numerous medical and psychiatric comorbidities, including chronic pain. Additionally, approximately 71% of patients with hEDS undergo at least one surgical procedure; however, indicators for surgery and pain outcomes after surgery are poorly understood. This preliminary study used a medical chart review to identify the frequency and nature of comorbidities in a cohort of adolescents and young adult patients with hEDS and a surgical history compared to those without a surgical history. Results showed that patients diagnosed with hEDS who underwent surgery reported significantly more comorbidities (e.g., CRPS, IBS, Fibromyalgia, POTS, hypothyroidism, etc.) than those who did not have surgery. Seventy percent of individuals who presented for surgery fell within the categories of orthopedic, gastrointestinal, or laparoscopic/endometriosis-related surgeries. Identifying patients with hEDS who are at risk for needing surgery will help identify the mechanisms contributing to risk factors for poor surgical outcomes. The results of this study may be instructive in the management and care of hEDS patients undergoing surgery.

## 1. Introduction

Hypermobile Ehlers–Danlos syndrome (hEDS), a subtype of Ehlers–Danlos syndrome (EDS), is one of the most common hereditary connective tissue disorders. A disorder such as EDS can impact the function of connective tissues in the skin, tendons, ligaments, blood vessels, internal organs, and bones [1]. To date, hEDS has been classified as an inherited, autosomal dominant disorder with inheritance patterns present in families. Despite this knowledge, the genetic etiology of hEDS is unknown. Prevalence rates are estimated to be 80–90% of all EDS cases, thus predicting its prevalence to be 1/5000 individuals, representing 1–3% of the general population [1,2].

hEDS was previously characterized by its cardinal manifestations, including marked articular hypermobility, moderate dermal hyperextensibility, and minimal scarring [2]. Over time, our understanding of this rare disease has evolved to include symptoms of chronic pain, chronic fatigue, dysautonomia, and anxiety [1] The primary manifestations are musculoskeletal and involve generalized joint hypermobility. Despite its classification as a connective tissue disorder, hEDS lacks cutaneous factors and presents with mild skin involvement compared to other types of EDS such as vascular or classical subtypes [1,2].

The hEDS subtype has been further classified as a chronic pain syndrome. It exhibits many diagnostic overlaps with comorbidities such as irritable bowel syndrome (IBS), temporomandibular joint (TMJ) disorder, sleep disturbances, chronic fatigue, and psychiatric disorders. Commonly seen pathologies of hEDS include soft, stretchy skin that usually features no skin fragility. In addition, individuals often suffer from multiple joint dislocations and subluxations in the knees, hips, ankles, wrists, and jaw. Also, hEDS is comorbid with multiple medical and psychiatric diseases, further complicating this rare disease’s course and treatment [1,2]. As frequently seen in other chronic pain conditions, anxiety and depression are common in patients with hEDS. Although chronic musculoskeletal pain and visceral pain are common, other comorbidities include gastrointestinal and gynecological diseases [1,2]. 

Comparable to many other diseases involving musculoskeletal pain, the most successful treatments of hEDS are multidisciplinary and include a management program consisting of a mix of medications, physical therapy, behavioral interventions such as cognitive behavioral therapy, lifestyle adjustments, and bracing for joint stability. In unique situations, pain-modulating drugs such as tramadol, opioids, tricyclic antidepressants (TCAs), selective serotonin reuptake inhibitors (SSRIs), or cyclooxygenase-2 (COX-2) inhibitors are prescribed. [3]. In many cases, surgical options are also frequently pursued. Studies have shown that the highest prevalence of surgeries are performed on the upper and lower extremities. This is expected considering hEDS is a musculoskeletal disorder often presenting with high rates of subluxations and joint pain [1,2]. However, little is known about surgical outcomes in these patients and how comorbidities may impact surgical course and recovery. This is particularly problematic given that presurgical pain, such as with a rare disease like hEDS, predicts chronic postsurgical pain [4], which can result in further disability.

The present study is a retrospective chart review of 100 adolescent and young adult patients diagnosed with hEDS in order to better understand the demographic characteristics and comorbidities in young patients presenting for surgery compared to those without a surgical history. We focused on two issues: (1) To quantify the number of surgical procedures that this cohort of adolescent and young adult patients has undergone and to examine the prevalence of specific surgical procedures in this sample. We hypothesized that while orthopedic surgeries would be prevalent given the nature of hEDS and joint subluxations, due to the prevalence of comorbidities, we would also find a history of surgeries impacting multiple organ systems. (2) To describe the demographic characteristics and comorbidities to better characterize who with hEDS typically presents for surgery versus those who do not require surgery. It was hypothesized that those in the surgical group would have more medical and psychiatric comorbidities compared to the non-surgical group. Identifying patients with hEDS who are at risk for needing surgery or multiple surgeries will help to elucidate the mechanisms contributing to risk factors for poor surgical outcomes, including treatment-refractory chronic pain, and help to inform a personalized medicine approach in the pre-, intra-, and postoperative phases.

## 2. Materials and Methods

### 2.1. Patient Group and Inclusion Criteria

For the characterization of a cohort of individuals with hEDS, the medical records of patients diagnosed with EDS receiving care at Boston Children’s Hospital (BCH) between the dates of 1 October 2019 and 10 February 2021 were obtained. Patient charts were viewed via an electronic medical record (Powerchart) following Boston Children’s Hospital Institutional Review Board ethics approval (IRB protocol number: IRB-P00035177; approved on 16 April 2021). The first 100 medical records were randomly selected and screened for this retrospective chart review. All extracted data and related files were stored on the research lab’s password-protected drive, which was developed in close collaboration with BCH Research Computing. The medical record number (MRN) was temporarily collected for each chart in one column of the data extraction tool on the drive, and each record was assigned a study identification number (ID) unique to the chart review. After the data had been extracted from each chart in this sample and every record assigned a study ID, all MRNs were deleted from the data extraction tool.

**Figure 1:**
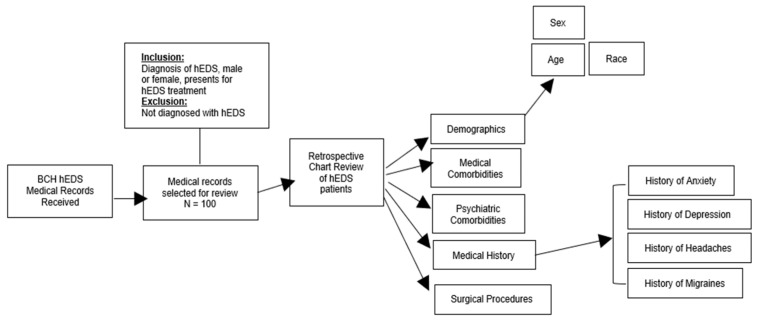
Experimental workflow for retrospective chart review. Figure 1 visually describes the experimental workflow that was utilized during this review. The medical records selected for review (*n* = 100) were chosen randomly only after the patients were confirmed to have a formal hypermobile Ehlers–Danlos syndrome (hEDS) diagnosis. Variables of interest for the review were extracted from physician clinic notes documented in Powerchart electronic medical records at Boston Children’s Hospital (BCH).

The inclusion criteria for this review included male and female patients who presented for treatment with a formal diagnosis of hEDS (Figure 1). Exclusion criteria included patients who lacked a formal diagnosis of hEDS. There was no formal consent needed for this study. Data assessed and evaluated in this analysis were as follows:

(1) Basic demographics, including age, sex, race, and sex.
(2) Associated comorbidities/experienced symptoms, including frequency of medical comorbidities (not typical symptoms of hEDS), frequency of psychiatric comorbidities, history of anxiety or depression, and history of migraines or headaches; and
(3) Surgical history, including frequency of each surgical procedure type.

### 2.2. Analysis

Analyses were completed using IBM SPSS Version 27.0.1. Descriptive statistics including frequencies and means were analyzed for demographics including age, sex, and race. Frequencies were also found for each participant’s medical comorbidities, psychiatric comorbidities, and surgeries. In addition, the frequency of procedure types was also assessed. Each of these variables was classified as nominal and continuous. After finding the determined frequencies of each surgical procedure type, the presence or absence of surgical procedures was coded to assess and compute a new grouping variable. These grouping variables were organized as dichotomous, categorical variables (Yes = 1, No = 0). This variable was used to measure any overlap between surgical procedure types. For example, the number of individuals who underwent one orthopedic surgical procedure alone could be compared with the number of individuals who underwent both orthopedic and gastrointestinal procedures.

A test for normality was run to determine if this data was a normalized sample. Both the Kolmogorov–Smirnov test and Shapiro–Wilk test were used to reach the conclusion that this was a non-normally distributed sample. As a result, the non-parametric Mann–Whitney U test was used to assess differences between the groups. The first set of analyses utilized biological sex (male or female) as formally reported on Powerchart as a grouping variable to determine if the frequencies of medical and psychiatric comorbidities were statistically significant between sexes. The second set of analyses utilized the presence (Yes = 1) or the absence (No = 2) of surgical history as a grouping variable. Specifically, frequencies of medical comorbidities and psychiatric comorbidities were compared between the individuals who underwent surgical treatment and those who did not. All differences were considered statistically significant at a 5% probability level, and all reported *p*-values were two-sided. 

## 3. Results

### 3.1. Patient Sample

Among the total sample of 100 patients diagnosed with hEDS, the sample was largely female (80%) and White (89%) (Table 1). Since there was a significantly uneven distribution of males (20%) to females (80%) in this cohort, the question of differences between sexes was not pursued further as a grouping variable. Age at the time of chart review was calculated using the birth date and date of chart review. Ages ranged from 12 to 42 years old with a mean age of 20 years (SD = 4.6 years). Among the total sample, 38% were aged 18 and younger, and 62% were aged over 18. 

### 3.2. Comorbidities

The frequencies of classified medical and psychiatric comorbidities (see Table 2), unrelated to the hEDS diagnosis, were examined. The mean frequencies and standard deviations were calculated, and the results are displayed in Table 3. Of note, only one patient had no known medical comorbidities, whereas another patient had 21. The mean number of medical comorbidities was nearly 7 among the entire sample (mean = 6.6; SD = 4.6). Over half (59%) of the sample had documented psychiatric comorbidities, with the maximum number of reported psychiatric comorbidities being 4 for one patient. The mean number of psychiatric comorbidities was 1.2 (SD = 1.2). Of the 59% of the total sample of individuals reporting psychiatric comorbidities, 47% reported a diagnosis of anxiety and 30% reported a diagnosis of depression. Among the 95% of the sample reporting one or more medical comorbidities, 26% reported experiencing headaches and 28% reported experiencing migraines. 

The presence of surgical treatment for hEDS was used as a grouping variable (Table 4). Surgical (*n* = 77) and non-surgical (*n* = 22) groups were examined and compared to determine if presentation for surgery had any impact on the frequency of medical and psychiatric comorbidities. 

In the surgical group with one or more reported surgical procedures, an average of seven medical comorbidities were reported (SD = 4.7). In the non-surgical group, an average of five medical comorbidities were reported (SD = 4.2). The surgical group reported an average of 1.2 psychiatric comorbidities (SD = 1.2), whereas the non-surgical group reported an average of 0.8 psychiatric comorbidities (SD = 1.0). Using the Mann–Whitney U test as a nonparametric method of analysis, the comparison of medical comorbidity frequencies in the surgical group with the non-surgical group was found to be significant, *p* < 0.05. Again, by using the Mann–Whitney U test to compare the frequencies of psychiatric comorbidities in the surgical group with the non-surgical group, no statistical significance was found.

**Figure 2:**
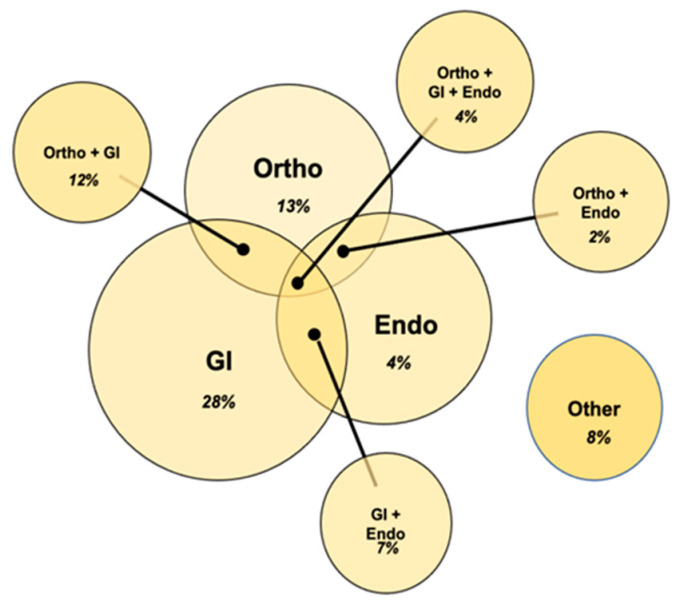
Representation of surgical procedure type. This diagram shows the types and numbers of surgical procedures indicated for the total sample of hEDS patients (*n* = 100) in the retrospective chart review. Endo = laparoscopic/endometriosis-related procedure; GI = upper/lower gastrointestinal procedure; hEDS = hypermobile Ehlers–Danlos syndrome; Ortho = orthopedic procedure.

The surgery procedure type was assessed among the surgical group. The three types of surgical procedures that this cohort underwent most frequently were orthopedic surgical procedures (Ortho), upper/lower gastrointestinal procedures (GI), and laparoscopic/endometriosis-related procedures (Endo). Table 5 reports the results found when comparing frequencies of each procedure type among this total surgical sample. Based on these results, 13% of patients were found to have orthopedic procedures only, 28% of patients were found to have GI procedures only, and 4% of patients were found to have laparoscopic/endometriosis-related procedures. The frequencies of surgical procedure type overlap are also reported in Table 5. Overall, 70% of this cohort underwent surgeries that fell within these three surgical procedure categories. The other 30% of this cohort underwent surgeries that did not fall into these categories, including bariatric surgery, endocrine surgery, head and neck surgery, and general surgery. Of this sample, 25% underwent more than one of these surgical procedure types, whereas 45% underwent only one. Figure 2 further highlights these frequencies.

## 4. Discussion 

In this preliminary study, we compared the frequency of medical and psychiatric comorbidities present in a cohort of young patients diagnosed with hEDS who had a surgical history compared to those with hEDS without a surgical history. In addition, types of surgical procedures were evaluated to assess the prevalence of certain types of surgery and to understand whether there were any common trends in the type of surgery performed and frequency of surgery. The initial hypothesis that surgical treatment would be associated with patients with more frequent comorbidities was supported. In addition, three types of surgical procedures were found to be most prevalent among this population: orthopedic surgery, gastrointestinal surgery, and laparoscopic/endometriosis surgery. While it was hypothesized that the most common procedure type would be orthopedic based on the nature of hEDS as a hereditary connective tissue disorder causing joint hypermobility, it was found that upper/lower gastrointestinal (GI) procedures were actually the most frequent. Specifically, 28% of individuals in this cohort received only GI procedures and no orthopedic procedures. In addition, when reviewing comorbidities in relation to surgical treatments pursued, many individuals suffered from pain profiles that consisted mainly of GI symptoms as noted in Table 2. 

Of this total sample, 25% underwent more than one surgical procedure, and 51% of individuals experienced at least one GI-related surgical procedure. Additionally, one of the most prominent findings was that in addition to hEDS symptoms, there was a high frequency of comorbidities spanning multiple body systems. Encompassing many body symptoms and varying between individuals, some of the most common comorbidities were postural orthostatic tachycardia syndrome (POTS), complex regional pain syndrome (CRPS), irritable bowel syndrome (IBS), gastroesophageal reflux disease (GERD), temporomandibular disorders (TMD), migraines, nausea, polycystic ovarian syndrome (PCOS), endometriosis, mitral valve prolapse, and asthma. Many of these comorbidities were expected based on the previous literature [1,2,3]. However, it is interesting to note that the multifocal pain associated with hEDS, and these comorbidities were not limited to one body system. Psychiatric comorbidities were also prevalent in this sample. Over half of this population, specifically 59%, reported one or more psychiatric comorbidities. These results highlight that this EDS subtype greatly impacts those affected, and the presence of additional multifocal pain might contribute to the difficulties associated with proper diagnosis and treatment. There were no significant differences found between the biological sexes when comparing the frequency of comorbidities or the frequency of surgical treatment. However, this should be further explored as data on differences between biological sex as it relates to hEDS is unknown.

One of the most significant findings from this preliminary data was found when examining comorbidities between groups of those who pursued surgical treatment and those who did not. A majority of individuals in this sample (77%) underwent one or more surgical procedure that fell into the three main surgery types including laparoscopy for endometriosis, upper/lower gastrointestinal procedures, and orthopedic surgeries. Of those who underwent at least one surgical procedure and were classified as part of the surgical group, there was a significantly higher number of medical comorbidities compared with the group having no surgical history. This finding supports the need for personalized treatment based on each patient’s presentation of this rare disease. 

### Future Directions

It is crucial to investigate the demographic, medical, and psychiatric profiles of patients with hEDS presenting for surgery. hEDS is a rare disease that can have detrimental physical and emotional consequences on a person, and as a result, this syndrome can significantly impact one’s quality of life. One consequence of adolescents and young adults living with this disease is choosing which treatment modality best relieves the diverse manifestations and symptoms of hEDS and produces the most positive outcomes. According to Rombaut et al. [5], in a cross-sectional study of 79 individuals diagnosed with hEDS and seeking treatment, 71% pursued a surgical treatment option. Despite this high percentage, not every patient with hEDS undergoes surgery, and some may choose to utilize medications, physical therapy, and behavioral treatment. However, there is minimal evidence documenting which modality brings the most relief to a patient and surgery may not always relieve the symptoms, especially pain, of hEDS. Research on short- and long-term outcomes of surgical intervention for people with hEDS compared to other treatment modalities is lacking. Specifically, systemic evaluation of chronic pain in patients with rare diseases is needed. Sieberg et al. [6] proposed a model evaluating the peri-surgical process, which involves identifying problems and solutions within the pre-, intra-, and postoperative pain states in order to both prevent the transition from acute to chronic pain, as well as prevent the development of long-term pain. For patients with hEDS, it is critical to evaluate whether surgery is truly the most optimal method for the treatment of the chronic pain associated with this disease, and if so, then careful monitoring needs to occur [6].

Further research is also warranted on whether individuals with hEDS who have more severe chronic pain or who suffer from multiple comorbidities are more likely to seek a surgical treatment option. Additionally, repeated exposure to chronic pain, as is the case with hEDS, likely results in allostatic load, which progressively leads to maladaptive brain plasticity, resulting in the chronicization of pain [7]. This concept is supported by the correlation observed between structural and functional abnormalities in the brain and disease severity measures among patients with chronic pain [6,7]. It is likely that pain cognitions and pain-related worry, as well as factors such as stress, play a role in modulating nociceptive processing in patients with chronic pain; pain-related brain activity may vary among individuals based on their pain cognitions and emotional functioning. However, the specific relationship between these variables and hEDS and how this may confer risk for the need for repeated surgical interventions has not been explored and warrants further investigation. Similarly, there is a need to examine how key psychosocial constructs such as resiliency and quality of life may impact pain in this population, as well as postsurgical outcomes, and is an important area of investigation.

Although this study offers original findings on the clinical presentation of young patients with hEDS presenting for surgery, there are several limitations. First, this is a small sample limited to one pediatric hospital, making it potentially difficult to generalize findings. Second, complicating generalizability was that this sample was predominantly female and White. Third, the individuals placed in the surgical group and non-surgical group were not treated exclusively by surgery. Both groups included individuals treated by other modalities such as medication and physical therapy. These treatment methods were not considered as grouping variables for the data analysis. Additionally, we did not account for the use of medications used to treat the comorbidities in this sample. It is unknown whether or not certain medications altered the manifestation of comorbidities and/or pain presented. Lastly, we did not have information on short- and long-term surgical complications, which could potentially be an important factor in the need for additional surgeries in patients with hEDS and an important area of future investigation.

Given that the genetic etiology of hEDS has not been discovered, it is important to create a clinical profile for practitioners to follow, which can assist in leading to early disease detection and diagnosis. Based on a review of the current literature, it is apparent that there are gaps in research surrounding hEDS in adolescents and young adults. It is crucial that this population be studied further, and a major goal going forward should be to pursue evidence-based treatments and pain management recommendations by encouraging more comprehensive assessments and documentation of the disease course in individuals with hEDS. In addition, one specific goal should be to create validated guidelines for the management of EDS-related GI symptoms, especially considering the co-occurrence of these symptoms found in this cohort. These steps will help to better understand hEDS and will help in the long run to develop improved therapeutics and techniques for treating patients with this disease.

## 5. Conclusions 

Via data extraction, this preliminary study attempted to further describe profiles of a cohort of individuals with hEDS. Although these results helped to characterize the pain profiles, especially those presenting for surgery, of a cohort of adolescent and young adult patients with hEDS, there is an urgent need to further elucidate the complex biopsychosocial factors contributing to the common comorbidities and risk for surgery; this will ultimately result in an improved understanding of the disease, earlier identification and treatment of comorbidities, more informed presurgical preparation, and postsurgical care. Future directions should aim to advance what is known about this disease to improve the quality of life of individuals diagnosed with hEDS.

## Figures and Tables

**Table 1:** Demographics.

Demographic	Value	N = 100	Percentage
Biological Sex	Male	20	20
	Fe male	80	80
Age	<18 years old	38	38
	>18 years old	62	62
Race	White	89	89
	Asian	2	2
	Black	2	2
	Other	1	1
	Not Reported	6	6

**Table 2:** Comorbidities found in cohort (not including traditional hEDS symptoms). This table shows all comorbidities recorded in the cohort of patients diagnosed with hEDS following the retrospective chart review.

Comorbidity Type	System	
**Medical**	Digestive	Irritable Bowel Syndrome (IBS), Gastroesophageal reflux disease (GERD), Nausea
	Chronic condition	CRPS, PCOS, Endometriosis, chronic kidney disease, scoliosis, fibromyalgia
	Respiratory	Asthma, allergic rhinitis
	Endocrine/Metabolic	Hypothyroidism, obesity, vitamin D deficiency
**Psychiatric**	Psychological/Unspecified	Anxiety, depression, ADHD, eating disorders, autism spectrum disorder

**Table 3:** Frequency of comorbidities.

	Frequency of Medical Comorbidities	Frequency of Psychiatric Comorbidities
Mean	6.6	1.2
N	99	100
Std. Deviation	4.6	1.2

**Table 4:** Frequency of medical comorbidities in comparison to surgical treatment performed. The Mann–Whitney U test indicated that the frequency of medical comorbidities is greater for individuals who underwent surgical treatment (mean rank = 53.2) than for those who did not (mean rank = 38.9).

Frequency of Medical Comorbidities	Surgical Treatment	N	Mean Rank	Sum of Ranks
	Yes	77	53.2	4093.5
	No	22	38.9	856.5
Total		99		

**Table 5:** Surgical procedure type quantification. This table shows the frequencies of specific surgical procedures in the surgical group compared with the total sample of hEDS patients (*n* = 100) in the retrospective chart review. Three types of surgical procedures are included in this overview: laparoscopic/endometriosis-related procedure (Endo), upper/lower gastrointestinal procedure (GI), and orthopedic procedure (Ortho). Twenty-two individuals underwent no surgical procedures, and eight individuals underwent surgical procedures that did not fall into the three reported categories.

Surgical Procedure Type	N	Percentage
Non-Surgical	22	22
Ortho Only	13	13
GI Only	28	28
Endo Only	4	4
Ortho + GI	12	12
Ortho + Endo	2	2
GI + Endo	7	7
Ortho + GI + Endo	4	4
Other	8	8

## Data Availability

No new data was generated as this was data extracted from the medical records so there is not a publicly available dataset.
